# On Some of the Unusual Effects of Atropine, and the Means to Counteract Them

**Published:** 1879-01

**Authors:** F. C. Hotz


### Article IV.

### On Some of the Unusual Effects of Atropine, and the Means to Counteract them. By F. C. Hotz, m.d. (Read before the Chicago Medical Society, Dec. 2, 1878.)

The form in which atropine is generally used in ophthalmic practice, is a watery solution of sulphate of atropia (0.1 in 25.0 grams). A drop of this solution put upon the conjunctival surface of the lower lid causes no pain, and no more irritation than a drop of pure water. As a rule, it has this pleasant effect upon healthy eyes as well as upon those affected with the inflammations for which the use of atropine is indicated.

This rule, however, applies only to the use of pure sulphate of atropia, which makes a clear, colorless, neutral solution, without any sediment. The presence of free acid, sediments, or any other impurity, manifests itself quickly by an unpleasant effect upon the eye. And as it occasionally happens that an inferior article is dispensed by druggists, it may be well to know that the eye is an exceedingly sensitive test-object for the chemical purity of atropine, and that by its reaction we may sometimes prove a solution to be adulterated which in appearance seems to be quite the reverse.

In August I prescribed atropine to a young lady for acute ulcerative keratitis. Two days afterward, when she called again, I was surprised to find the pupil undilated, although the iris was not implicated in the morbid process, and the corneal inflammation of such a degree that I expected a quick and complete mydriasis. As patients and their attendants are often exceedingly awkward in the manipulation of eye-drops, I attributed the absence of mydriasis to a failure on the part of the patient in applying the medicine. I instilled a drop of a one per cent. solution I kept at my office, in order to show her how it should be applied. The pupil readily responded to my atropine, and was completely dilated when the patient left the office. Still again, after two days, she came with her eye unimproved and the pupil undilated; but she assured me that the drops had been used regularly, and that she was sure they had reached her eye; she said she could feel them, for every time the drop was put in her eye, it smarted for about fifteen minutes. This statement was sufficient evidence that the medicine had been applied properly, and if it had not the desired effect, the failure could not have resulted from insufficient use, but was from an abnormal action of the medicine itself. And it remained to be determined whether this anomalous effect was produced by an impure solution or by a peculiar intolerance of atropine on the part of the eye. The solution the lady showed me was clear, colorless, free of acid and sediment, had the characteristic taste of atropine; in short, it seemed to differ in no way from my solution, which I knew to be perfectly pure and trustworthy. But when I put a drop of my solution in the lady's eye, it did not cause any smarting, the pupil soon began to dilate, and under the continued use of my solution the pupil remained dilated, and the eye quickly recovered. This experiment showed that there was something wrong with the other solution. For if the smarting it produced had been due to an intolerance of atropine on the part of the eye, the same unpleasant effect would have succeeded the instillation of my solution; but if the solution of known purity acted normally in every respect, while the other produced abnormal symptoms, we must logically infer that these symptoms were due to an adulteration of the atropine in the second solution.

I relate this instance because it may show that in cases in which we believe we have had an anomalous local effect of atropine, we must always ascertain whether the atropine employed was pure. An impure alkaloid will always act as an irritant upon the eye, while the pure atropine sulphate does so in very exceptional cases.

But occasionally we meet with patients upon whom the use of the purest alkaloid has a peculiar effect, altogether different from that which we are accustomed to observe. In all these cases, I believe, there exists a peculiar susceptibility of the patients, which manifests itself by an unusual, local or constitutional effect, of the drug.

The local idiosyncrasy—if I may use this phrase—is characterized by a sense of smarting and heat in the eye, accompanied by redness of the conjunctiva and an increased secretion of tears the first and every subsequent time atropine is dropped in the eye. These symptoms may pass off in a few minutes, or they may last a few hours.

As an illustration of this kind of unusual effect of atropine, I will cite the following observation.

In May, 1877, a gentleman consulted me in regard to an increasing dimness of his left eye. The pupil was very small, and to dilate it for the purpose of an ophthalmoscopic examination, I instilled a drop of my atropine solution. As it had acted very pleasantly on all previous occasions, I was surprised to hear this patient complain of considerable smarting after the atropine was dropped in the eye. The pain was so great that the patient had to keep his eye shut for a few minutes, and when he was able to open it again, the conjunctiva was very red, and the tears ran over the lower lid freely. But the pupil became fully dilated in due time, and I could ascertain that the fundus of the eye was normal, and the impairment of vision was caused by a slight central cloudiness of the crystalline lens. At that time it did not occur to me that there was any relation between the peculiar symptoms which followed the use of atropine, and the drug itself, but I regarded their occurrence as merely incidental. But in August the patient came back for a second examination, and the use of atropine produced the same smarting and redness in the eye as I observed on the first occasion. And then it struck me that between the atropine and these symptoms there was a relation of cause and effect, and that this eye was unusually intolerant of the solution.

But the most remarkable instances of atropine idiosyncrasy are those cases in which a single drop of atropine put in the eye produces characteristic symptoms of constitutional disturbance. A short time after the application of atropine (which may or may not cause a slight smarting in the eye), the patient is taken with palpitation of the heart, precordial anxiety, headache, dryness of the throat, and restlessness which is considerably aggravated by a burning and intolerable itching all over his body. The face then becomes flushed, as if the patient had been standing over a hot stove, or was in the fever heat of intense excitement. This redness swiftly extends from the face to the neck, arms and over the other parts of the body, so that in half an hour the patient looks as if he had scarlet fever. These symptoms subside in the course of a few hours; sometimes desquamation of the epidermis succeeds this scarlatinous eruption.

Cases of this unusual susceptibility for atropine have been frequently observed in ophthalmic and other practice. So we find in Ziemssen's Cyclopædia, vol. xvii, p. 667, the following instances: "J. G. Wilson (*Glasgow Med. Journal*, Feb., 1872) saw two cases in lying-in women who had belladonna ointment rubbed on their breasts to produce galactostasia, and upon whom a scarlet eruption came out, which lasted three or four days. Stadler (*Med. Times and Gaz.*, April, 1868) saw a similar eruption appear upon a child three months old, within a few minutes after the administration of $\frac{1}{210}$ grain (0,0003) of atropine sulphate for whooping cough. It lasted five hours."

If similar cases are oftener observed by ophthalmic surgeons than by practitioners, it is because they use atropine so much more frequently. The symptoms of intoxication are the same whether the alkaloid is given internally or applied externally; but it is interesting to consider the infinite smallness of the dose which is sufficient for the production of a toxic effect in those cases. One drop of a half per cent. solution contains about 0.0003 ($\frac{1}{240}$ grain) of atropine; but it is safe to assume that at least one half of this drop is washed out by the tears and runs over the lower lid; and only 0.00015 ($\frac{1}{480}$ grain) remains to be absorbed by the cornea and conjunctiva, and taken up into the blood causes the constitutional disturbance.

It is the unanimous opinion of all writers that when a patient exhibits symptoms of idiosyncrasy, the further use of atropine should be dispensed with. Under circumstances, however, this advice may place the attending physician in a very embarrassing position; for instance, if he has to treat a case of acute iritis. If he does not use atropine (and he cannot obtain any other reliable mydriatic) the pupil remains contracted, and probably will be occluded by circular synechiæ; if he does use it, his patient will be in a state of continuous intoxication. Should he suffer the eye to be permanently damaged, or subject the patient to the annoyance and danger of atropine poisoning? This is a serious dilemma; and the only satisfactory solution seems to me is to find some remedy which possesses the property of counteracting the unpleasant effects attending the use of atropine, without interfering with its valuable local influence upon the eye.

The antagonism of action of atropine and morphine is a well established fact and has been utilized by many in counteracting the nauseating effect hypodermic injections of morphine produce in many patients, by adding to the morphine solution a small amount of atropine. It has also been proven, in a number of instances, by the antidotal effect hypodermic injections of morphine had in poisoning by atropine and vice versa. And the hypodermic use of morphine was also recommended by Graefe and others to counteract the constitutional effect sometimes produced by the local use of atropine. These considerations led me to try whether the unpleasant constitutional effect of atropine could not be neutralized or altogether obviated by adding a little morphine to the atropine solution intended for local use. During last summer I tried this experiment in the following case, which exhibited the typical signs of idiosyncrasy after each instillation of atropine.

### Acute Ulcerative Keratitis; Atropine Idiosyncracy Relieved by Morphine.

Mrs. C. S., aged 39 years, of a very nervous temperament, has repeatedly suffered from inflammations of her eyes. Thirteen years ago the right eye was the seat of a protracted and painful inflammation, which permanently impaired its sight. In March, 1873, I treated her left eye for marginal keratitis. She had several slight attacks since, but the eye always recovered in a few days. In June last, both eyes became very red and extremely painful. The patient, encouraged by previous experience, expected to get over this attack just as quickly; but after patiently waiting in vain three weeks for the change, she at last concluded to seek medical advice. I saw the lady on June 26, and found her very nervous and worried, particularly about her left eye which, she feared, would become as blind as her right one. There was a copious secretion of tears, marked intolerance of light, and great supra-orbital neuralgia. The eye-balls were tender to pressure, and showed a broad zone of pericorneal injection; in the left cornea, near the upper margin, a small, longitudinal, superficial ulcer; in the right cornea an old central cloudiness of the epithelium, but no new infiltration. The pupils were contracted, but dilated readily and regularly in the dark. I prescribed a half per cent. solution of sulphate of atropia, to be instilled into each eye three times daily; and a saline cathartic.

At my visit on the next day I found the eyes better and the pupils fully and regularly dilated; the patient had no pain, but she said that fifteen minutes after each application of the drops she had felt a great uneasiness, with palpitation of the heart, dryness of throat, a burning and itching sensation all over her body, and, soon after, her face and whole body were as red as if she had the scarlet fever. This lasted about one hour; then the integument recovered its natural color and the patient felt quite well.

The next day the patient gave the same report, and I could convince myself of the correctness of her observation. I instilled with the dropping tube one small drop of the atropine solution into the left eye, and had her keep the eye closed for a few minutes, while I was pressing upon the lachrymal sac. In fifteen minutes by the watch the acceleration of the heart's action manifested itself in the quickened pulse; the patient felt oppressed, as though she would suffocate; had that burning and intolerable prickling of the skin; her face became scarlet, and this redness swiftly extended over neck, hands and arms.

This observation left no doubt that these symptoms were the constitutional effect of the minute dose of atropine administered, and that the medicine had to be discontinued unless its constitutional effect could be prevented. I disliked the idea of abandoning the use of atropine, and it occurred to me to try whether the antagonism of morphine and atropine could not be utilized in this case. And in order to make the experiment conclusive, I had the same solution of atropia continued, but added to it 5 centigrams of morphiæ sulphas. The patient used this combination without any discomfort; those annoying constitutional disturbances did not once re-appear, and the eyes got well.

But in September the patient had a relapse of keratitis in the left eye. For the sake of testing her idiosyncracy again, I put one drop of a pure one-half per cent. solution of atropine into the eye. It had precisely the same effect as in June; and again, the addition of morphine arrested the constitutional effect, without interfering in the least with the local action of the atropine upon the iris.

It will be noticed that in the above described case the constitutional symptoms occurred even when I employed these precautionary measures which prevent the entrance of atropine into the tear passages and the pharynx, whence it is more rapidly absorbed than in the eye. Sometimes the constitutional effect can be obviated by pressing upon the lachrymal sac with the finger during and a little while after the instillation of atropine.

This measure is especially successful in those cases in which the absorption of such minute doses of atropine produces no other sign of poisoning than dryness in the throat. One of my patients, who was greatly annoyed by this dry feeling in the pharynx, operated in the following manner: When he put atropine in his right eye, he laid down and turned his head to the right, so that the tears would flow toward the external canthus, as the lowest part of the conjunctival sac. By thus reversing the tear current for a minute, the atropine which was not absorbed by the eye was washed out by the tears, and none got into the throat.

Under circumstances, the entrance of atropine into the pharynx produces more distressing symptoms than dryness of the throat. All along its passage over mucous membranes the atropine is eagerly absorbed, and although each drop contains but a very minute dose, a sufficient amount may gradually accumulate in the blood to produce marked toxic symptoms. Children have shown signs of poisoning after a few instillations of the half per cent. solutions; and in adults similar accidents have occurred if stronger solutions were used, and especially if the applications were made in short intervals. I remember very well a case which occurred in my practice some four years ago, and I will give a brief report of it from notes kindly furnished by Dr. W. T. Montgomery.

### CASE OF POISONING FROM LOCAL USE OF SOLUTION OF ATROPIA.

In March, 1874, Mr. A. K., aged 72 years, in good general health, had cataract extracted from each eye, Dr. Hotz performing the operations. The day after extraction, the wounds were found closed, and a solution of atropia sulphate, 0.1 to 40., was ordered to be dropped into the eyes three or four times daily. On the third day slight iritis was noticed, when a solution of 0.2 to 40. was ordered to be dropped into the eyes once in two hours. The day following this the patient began to be delirious, and to the delirium was soon added all manner of absurd hallucinations, so that the attendant became alarmed, and thought the patient was coming down with fever or some other grave disease. The pulse was found rapid and feeble, skin flushed and dry, tongue dry and brown, great thirst and dryness of throat. The temperature was taken and found to be normal. This fact, of course, excluded the question of fever or severe inflammatory trouble. The next thought was that the old gentlemen had gone crazy.

The following night the delirium and hallucinations continued, so that the patient had to be restrained to keep him in bed and from tearing the bandage from his eyes. It had occurred to the attendant that the symptoms were those of belladonna poisoning, and when the local use of atropine was discontinued, and morphine was given internally in liberal doses, the patient quickly recovered.

In concluding these observations, I wish to repeat that these and other anomalous effects of atropine occur comparatively very seldom if we consider the frequent use of this alkaloid in ophthalmic practice. And while it is certainly very well for us to know that these incidents happen occasionally, and to remember the means by which to obviate or counteract them; they should not discourage us in the least from using atropine wherever its employment is indicated. For we must never forget that these exceptional results are outweighed a hundred fold, nay a thousand fold, by the good service atropine has done and always will do to people afflicted with diseases of the eye.

---

Glycerin of the Oxide of Zinc. F. Pollet.

| | |
|---|---|
| Glycerine | 16 |
| Starch | 8 |
| Oxide of Zinc | 1 |

Mix.

For the dressing of rhagades and chronic inflammation of the anus. Rhagades, which are little fissured ulcerations, situated in the natural folds of the anus, or frequently at the base of, or in the angle formed by mucous patches, vegetations or condylomata, often yield to simple cleanliness, to dressings with aromatic wine, or light cauterizations with nitrate of silver. It is when they are rebellious to these means that recourse is had to the glycerine of zinc. In point of fact, in order to obtain a definitive cure, it is first of all necessary to dissipate the lesions which give rise to the rhagades, and of which they are only a complication.—*L' Union Médicale.*

